# Supplementary material for: Complexome profiling of the Chlamydomonas psb28 mutant reveals TEF5 as an early PSII assembly factor
Source: Plant Cell. 2025 Mar 17;37(6):koaf055. doi: 10.1093/plcell/koaf055 (PMC12164590; doi:10.1093/plcell/koaf055)
Supplement: koaf055_Supplementary_Data [file koaf055_supplementary_data.zip › psb28_interactive.zip!/plots/intensity/qualityPlots/peptides/RAB23_Cre06.g262850.t1.2.html]

### Cre06.g262850.t1.2

Trivial name: RAB23  
SeqCoverage [median]: 9.80  
Mass: 28.04 kDa  
  
 

| Sequence | MissedCleavage | Mass [Da] | wtIntensity | muIntensity | Score |
| --- | --- | --- | --- | --- | --- |
| AVVTPEEVEALAR | 0 | 1382.74 | 926.48 | 2240.06 | 87.91 |
|  |  |  | 926.5 | 2240.1 |  |
